# Sporadic Creutzfeldt-Jakob Disease With COVID-19 Infection: A Case Report

**DOI:** 10.7759/cureus.45757

**Published:** 2023-09-22

**Authors:** Harjinder Singh, Thomas Yura, Vivek Kak

**Affiliations:** 1 Internal Medicine, Henry Ford Jackson Hospital, Jackson, USA; 2 Internal Medicine, Michigan State University, East Lansing, USA; 3 Infectious Disease, Henry Ford Jackson Hospital, Jackson, USA

**Keywords:** prion, dementia, encephalopathy, creutzfeldt-jakob disease, covid-19

## Abstract

Creutzfeldt-Jakob disease (CJD) is a rare and rapidly fatal neurological disease. Diagnosis is made through clinical features, imaging, electroencephalography, and cerebrospinal fluid analysis. Sporadic CJD accounts for the majority of cases and occurs due to somatic mutation in the gene or random structural change in the prion protein. Coronavirus disease 2019 (COVID-19) is known to cause neurodegeneration, and CJD acceleration is hypothesized due to systemic inflammatory response and prion misfolding. We present a 70-year-old lady with rapidly progressing dementia diagnosed as CJD, with the onset coinciding with COVID-19 infection.

## Introduction

Prion diseases occur due to the conversion of normal prion protein (PrPc) from an alpha-helical shape to a beta-pleated infectious particle (PrPSc), leading to conformational change and replication in the surrounding prion proteins [1]. The hallmark of Creutzfeldt-Jakob disease (CJD) is rapidly progressing dementia with neurological findings such as myoclonus and visual disturbances leading to cortical blindness, ataxia, and akinetic mutism in the later stages of the disease [2]. Sporadic CJD (sCJD) is the most common subtype and occurs at a rate of roughly one to two cases per one million population per year [3]. Its onset is usually in the seventh decade of life, with a median time to death of five months [4].

This article was previously presented as a poster at the American College of Physicians, Michigan Chapter Meeting on October 15, 2022.

## Case presentation

A 70-year-old lady was brought by her family to the emergency department due to decreased responsiveness and confusion over the last four days. She had a cough, fever, and myalgia for that time and tested positive for COVID-19 through reverse transcription polymerase chain reaction (RT-PCR) two days ago. On evaluation, the patient had stable vital signs and required 2 liters/minute of oxygen via nasal cannula. On neurological exam, she had decreased alertness and orientation with receptive and expressive language deficits, delayed responsiveness, and increased processing time. Motor exam, sensory exam, and cranial nerve examination were within the normal limits.

For evaluation of encephalopathy, diffusion-weighted sequence Magnetic Resonance Imaging (MRI) was obtained, which showed restriction located over the left occipital and parietal cortical region. These findings were presumed to be non-specific, and based on the clinical presentation, the cause of encephalopathy was presumed to be COVID-19 infection. The patient received intravenous steroids and remdesivir for its management. Due to her continuing encephalopathy and confusion, the patient required assistance with daily living activities. She was evaluated by physical medicine and rehabilitation and was discharged to a skilled nursing facility. Before this admission, she was fully independent in all the activities of daily living (ADLs), driving, and managing bills. In the nursing facility, she started developing bizarre behavior and uncoordinated muscle movements over one month and subsequently became wheelchair bound. The patient was imminently seen in the Neurology clinic and was noted to have aphasia, myoclonus, and a blank stare. Due to this rapid neurologic decline, she was sent to the emergency room and hospitalized.

Lumbar puncture (Table 1) showed no discernable evidence of bacterial, viral, or fungal meningitis. The suspicion for autoimmune pathology was low, given the history and low immunoglobulin levels in the CSF. Serum antibodies against *Histoplasma*, *Blastomyces*, *Aspergillus*, and *Coccidoides* were checked and were negative. CSF studies for CJD workup were sent.

**Table 1:** Lumbar puncture results CSF: Cerebrospinal fluid, IgG: Immunoglobulin G, WBC: White Blood Cells, RBC: Red Blood Cells, PCR: Polymerase Chain Reaction, RT-QuIC: Real-Time Quaking-Induced Conversion

Lumbar puncture studies	Result	Normal range
Albumin (mg/dL)	53.4	0.0 - 35.0
IgG (mg/dL)	5.1	0.0 - 3.4
IgG synthesis rate (mg/day)	0.00	0.00 - 3.00
IgG/Albumin index	0.44	0.00 - 0.77
Oligoclonal bands	Negative	Negative
WBC (per cubic mm)	0	0-8
RBC (per cubic mm)	3	<1
Glucose (mg/dL)	109	50-80
Protein (mg/dL)	90	15-60
JC virus PCR	Not detected	Not detected
Cryptococcus antigen	negative	negative
Histoplasma antigen (ng/mL)	<0.2	<0.3
Fungitell (pg/mL)	<60	<60
Tau protein (pg/mL)	>20,000	0-1149
14-3-3 (AU/mL)	96757	<30 - 1999
RT-QulC	Positive	Negative

**Figure 1:**
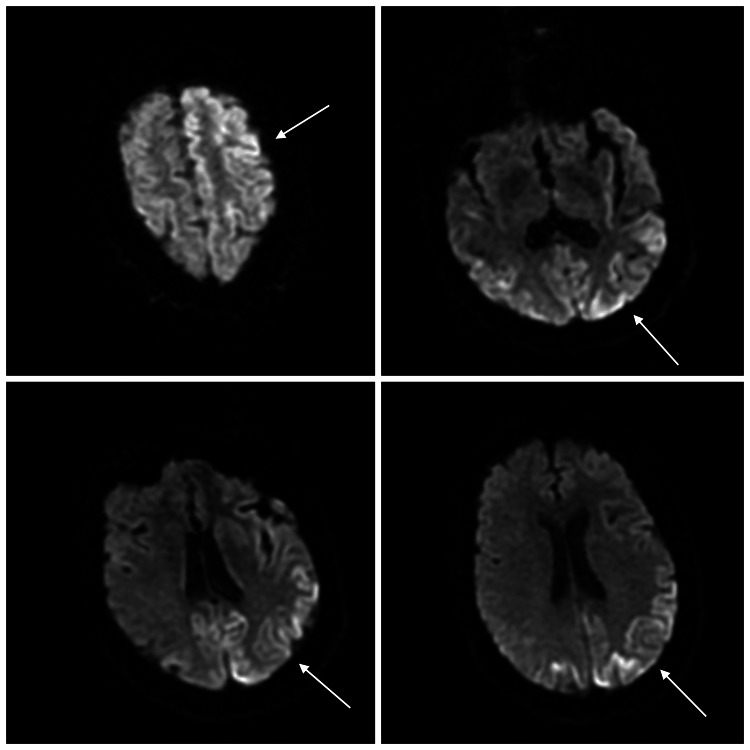
Diffusion-weighted sequence (DWI) MRI transverse section of brain showing gyral areas of restricted diffusion in left frontal, parietal, temporal, and occipital regions

Due to the rapid neurologic decline, an MRI-brain was performed again, which showed extensive cortical ribboning in the frontal and occipital cortex and gyral areas of restricted diffusion involving bilateral frontal, parietal, temporal, and occipital regions (Figure 1).

Electroencephalography showed triphasic periodic sharp wave complexes and generalized periodic discharges at 1 Hz, suggesting CJD. The workup of CJD from the lumbar puncture showed elevated Tau protein, elevated 14-3-3 protein, and a positive real-time quaking-induced conversion (RT-QuIC). The presence of positive RT-QuIC confirmed the diagnosis of CJD.

**Figure 2:**
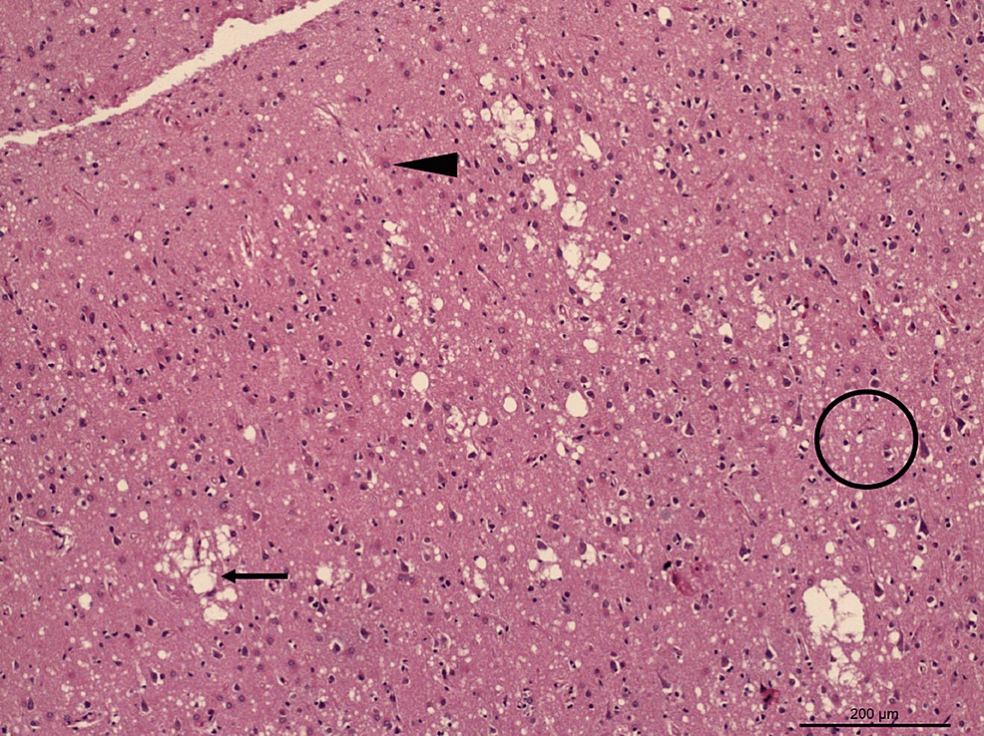
Brain (frontal cortex) biopsy showing vacuole spongiform degeneration (large arrow, small circle) and reactive astrocytes (arrowhead), hematoxylin & eosin

**Figure 3:**
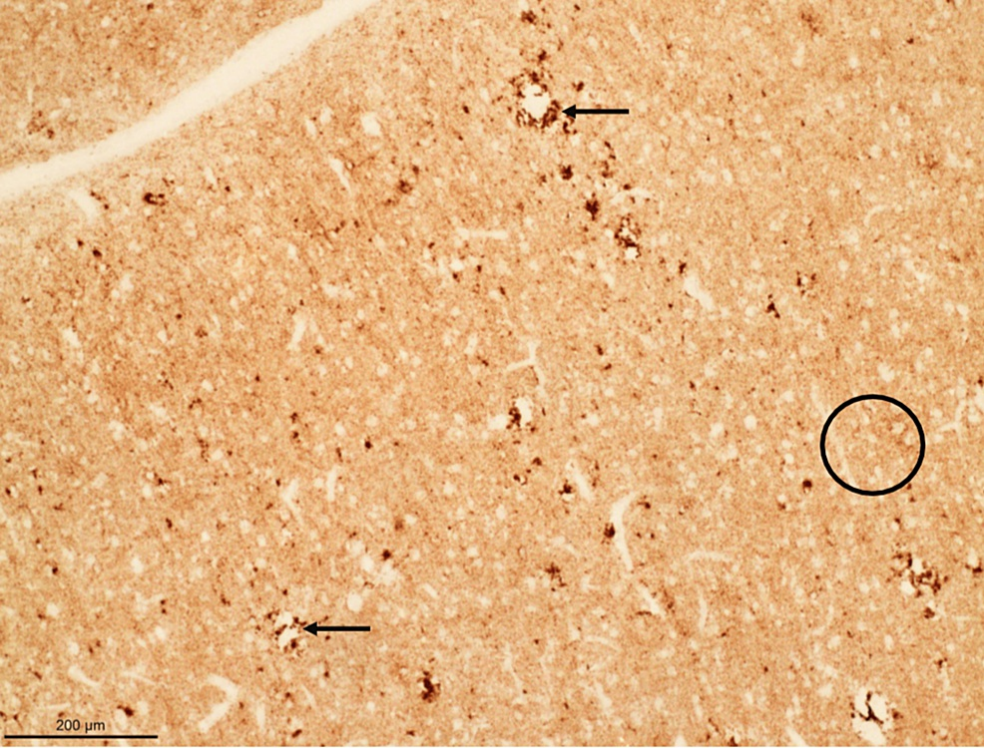
Brain (frontal cortex) with perivacuolar (arrows) and diffuse (circle) PrP immunostaining

**Figure 4:**
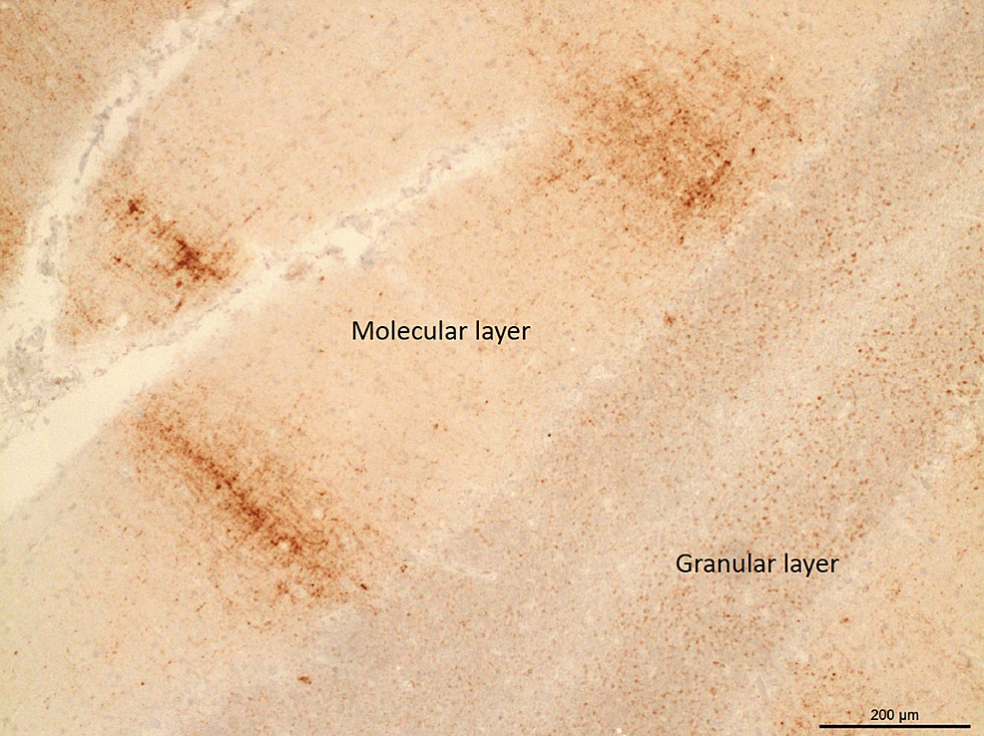
"Brush stroke-like" PrP immunostaining pattern in the cerebellum

The patient followed up with an infectious disease clinic, and the diagnosis of CJD was shared with her family. After discussing the prognosis and course of the disease, a decision was made to elect comfort measures with hospice. The patient passed away four months later. Post-mortem brain biopsy performed at the family’s request revealed evidence of spongiform degeneration (Figure 2) and a positive prion protein (PrP) immunostaining (Figures 3, 4) - western blot detected type 1 prion protein consistent with sporadic CJD.

 

## Discussion

sCJD is a rare neurodegenerative condition with a rapid and 100% mortality [1]. Due to the rapidity of progression, early detection remains paramount for early diagnosis. CSF 14-3-3 and CSF real-time quaking-induced conversion assay (RT-QuIC) are commonly used tests. The sensitivity and specificity of CSF 14-3-3 for diagnosing sCJD are 92% and 80%, respectively. Given the relatively low prevalence of sCJD, a specificity of 80% indicates a high false positive rate [5] and could be falsely elevated in conditions such as hypoxic brain injury, atypical encephalitis, metabolic encephalopathy, intracerebral metastases, and progressive dementia [6]. The RT-QuIC test detects pathologic prion proteins (PRPsc) using a recombinant prion protein (recPRP) as a substrate to demonstrate prion activity. The disease-causing prion protein induces a conformational change in the recPRP, which changes it to amyloid and facilitates its detection [7]. It has a sensitivity of 98.5%-100% and a specificity of 92%-95%. Because of its higher specificity, RT-QuIC is more appropriate for a disease with a low prevalence, such as sCJD [8]. This makes RT-QuIC the preferred confirmatory test for the diagnosis.

There are previously published case reports describing a relationship between infection with COVID-19 and the progression of neurodegenerative diseases. Young et al. [9] described a case of a 60-year-old male in whom CJD symptoms coincided with SARS-CoV-2 infection. That study hypothesized IL-1 and TNF release from COVID-19 infection driving extensive A1 astrocyte activation. That, in turn, is presumed to lead to the mediation of PrPSc production and subsequent development of CJD. Bernardini et al. [10] presented a case of a male in his early 40s who rapidly developed CJD two months after recovery from a mild COVID-19 infection. At the time of sCJD symptom onset, serum and CSF cytokine profiles exhibited high IL-6 and IL-8, consistent with central nervous system inflammation due to COVID-19 encephalopathy. The CSF samples also had increased CXCL10, indicating increased glial cell activation. The short interval between COVID-19 infection and neurological sequalae of CJD, along with persistent markers of COVID-induced inflammation, suggested a possible relationship between the systemic inflammatory response to SARS-CoV-2 and the rapid onset of CJD.

Another case reported by Olivo et al. [11] demonstrated concomitant COVID-19 pneumonia and a new onset of rapidly progressive dementia. That patient was found to have non-convulsive status epilepticus with further workup revealing positive RT-QuIC and cortical ribboning on MRI. There have been some proposed mechanisms through which COVID-19 can initiate or accelerate sCJD. In vivo models showed that the introduction of the SARS-CoV-2 spike S protein accelerates proteinopathic seeding of pathogenic tau aggregates. The models also showed that the interaction of the virus’ spike S protein with the angiotensin-converting enzyme-2 (ACE-2) receptor increases the cytosolic spread of prion and tau aggregates, leading to CJD [12].

Neurodegeneration is closely associated with aggregation of protein plaques in central nervous system tissue and is also shown to be associated with COVID-19 infection. The SARS-CoV-2 S1 spike protein may facilitate the aggregation of these plaques by interacting with several aggregation proteins with corresponding heparin-binding domains. These proteins include amyloid-beta, alpha-synuclein, tau, other prion proteins, and TDP-43. These amyloid proteins are thought to bind S1 on the viral surface and facilitate aggregation of these proteins by approximation, leading to the acceleration of neurodegenerative processes [13]. Both sCJD and COVID-19 induce a family of pathologic microRNAs (miRNA-146a-5p and miRNA-155-5p). Upregulation of these microRNAs (miRNA) is associated with severe systemic inflammation and progressive neurological disease. In addition to COVID-19, these miRNAs are induced by many other neurotropic DNA and RNA viruses. Early in the infectious course, expression of these miRNAs is thought to play a role in regulating the innate immune response, complement system, and inflammatory signaling in the CNS. This may point to a common mechanism underlying neurological dysfunction in both prion diseases and neurotropic infections [14].

## Conclusions

This case represents the association of sCJD with the onset of COVID-19 infection. There are hypothesized molecular pathogenesis of it leading to acceleration of sCJD. In the case of rapidly progressing dementia, early detection is essential for the limited duration of prognosis so that appropriate measures are taken to ensure necessary prognostication. This case also sheds light on the high specificity of the RT-QuIC test, which is confirmatory and could prevent the need for brain biopsy for diagnosis.
